# Supplementary material for: Female-enriched Eggerthella lenta drives neuroinflammation and IFN-γ via host receptor TLR2
Source: bioRxiv. 2026 Mar 23:2026.03.16.711194. Preprint. [Version 2] doi: 10.64898/2026.03.16.711194 (PMC13042044; doi:10.64898/2026.03.16.711194)

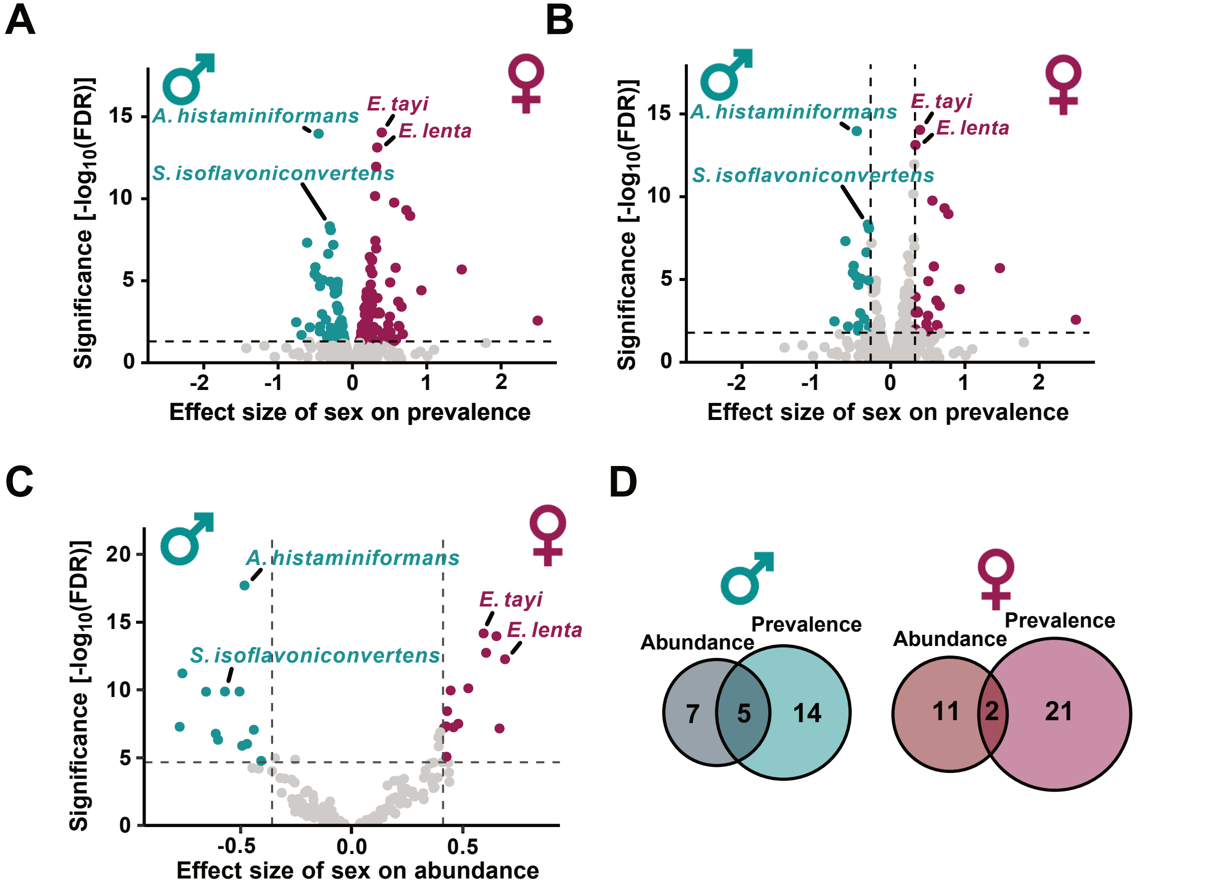

A
B
C
D
Significance [-log10(FDR)]
Effect size of sex on prevalence
Effect size of sex on abundance
A. histaminiformans
S. isoflavoniconvertens
E. tayi
E. lenta
Abundance
Prevalence
7
5
14
11
2
21

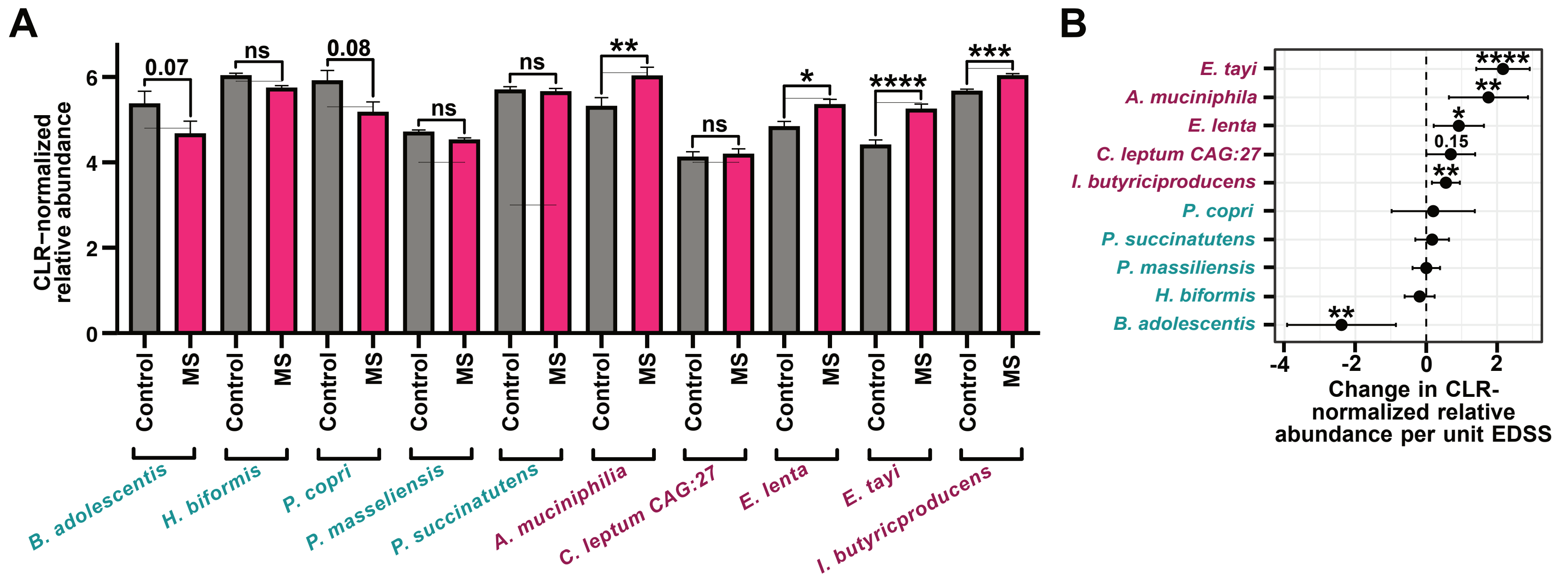

A
CLR−normalized relative abundance
0
2
4
6
0.07
ns
0.08
ns
ns
**
ns
*
****
***
Control
MS
B. adolescentis
H. biformis
P. copri
P. masseliensis
P. succinatutens
A. muciniphilia
C. leptum CAG:27
E. lenta
E. tayi
I. butyricproducens
B
E. tayi
A. muciniphila
E. lenta
C. leptum CAG:27
I. butyriciproducens
P. copri
P. succinatutens
P. massiliensis
H. biformis
B. adolescentis
****
**
*
0.15
**
**
-4
-2
0
2
Change in CLR-normalized relative abundance per unit EDSS

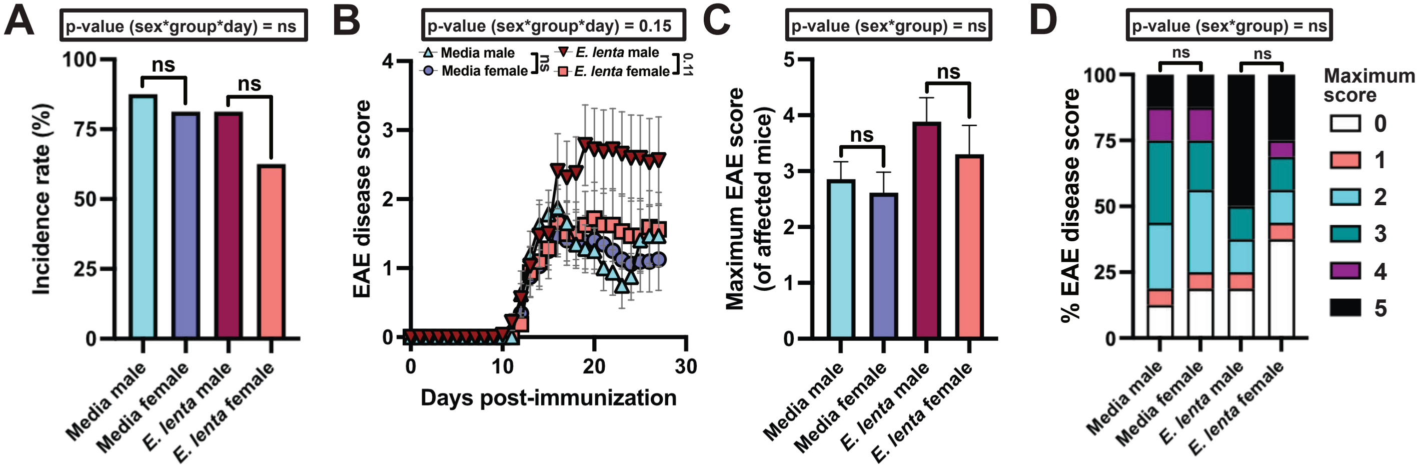

A
p-value (sex*group*day) = ns
Incidence rate (%)
ns
ns
Media male
Media female
E. lenta male
E. lenta female
B
p-value (sex*group*day) = 0.15
Media male
Media female
ns
E. lenta male
E. lenta female
0.11
EAE disease score
Days post-immunization
C
p-value (sex*group) = ns
Maximum EAE score (of affected mice)
ns
ns
Media male
Media female
E. lenta male
E. lenta female
D
p-value (sex*group) = ns
% EAE disease score
ns
ns
Maximum score
0
1
2
3
4
5
Media male
Media female
E. lenta male
E. lenta female

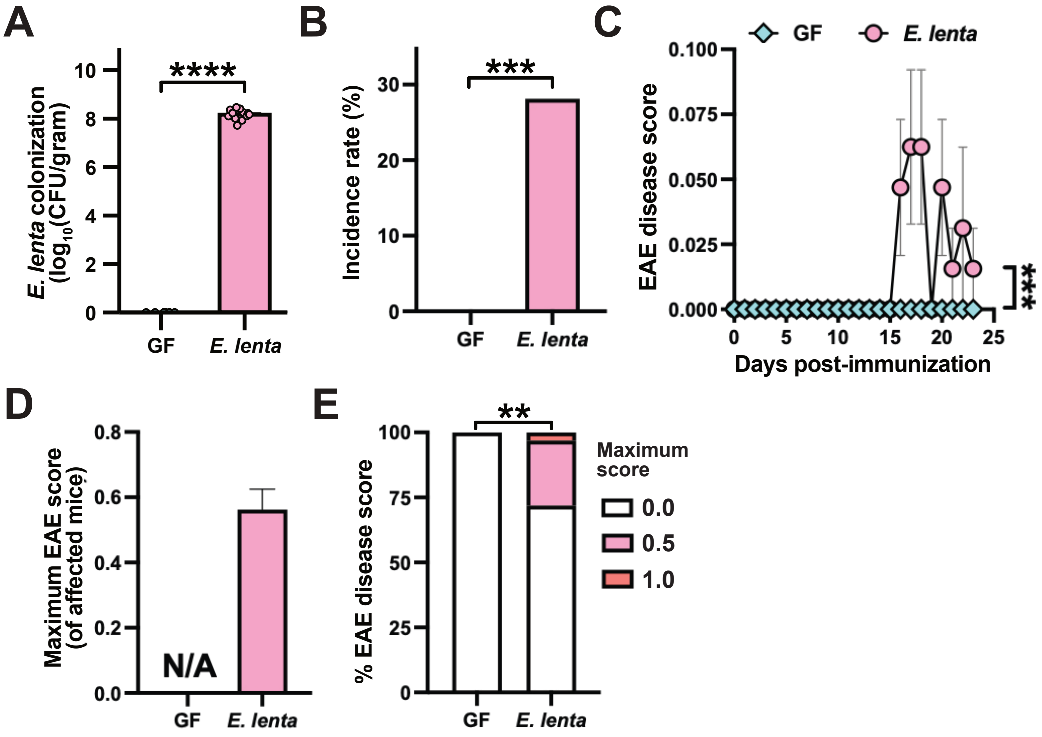

A
E. lenta colonization (log10(CFU/gram)
****
GF
E. lenta
B
Incidence rate (%)
***
GF
E. lenta
C
GF
E. lenta
EAE disease score
Days post-immunization
***
D
Maximum EAE score (of affected mice)
N/A
GF
E. lenta
E
% EAE disease score
**
GF
E. lenta
Maximum score
0.0
0.5
1.0

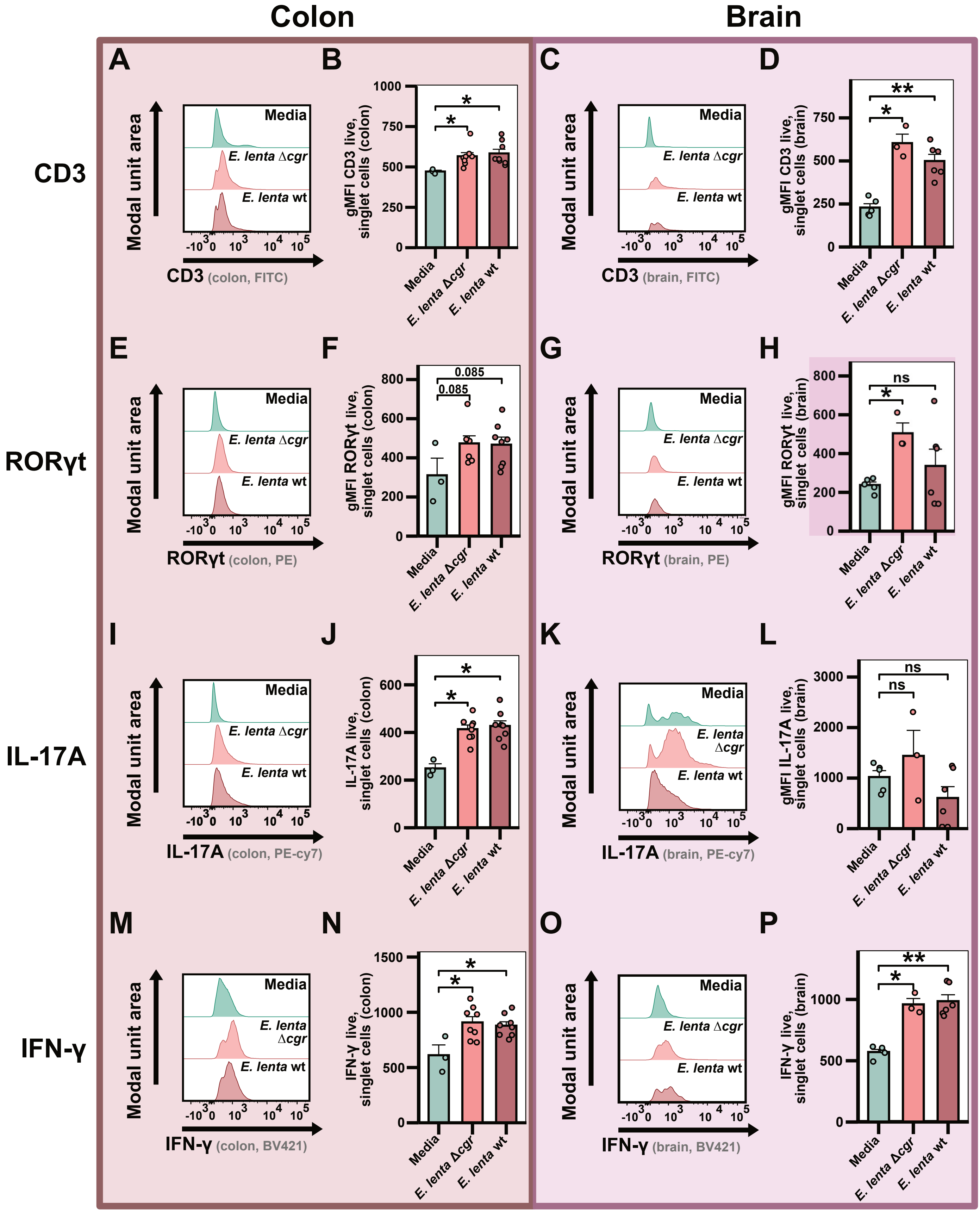

Colon
Brain
A
B
C
D
CD3
Modal unit area
Media
E. lenta Δcgr
E. lenta wt
CD3 (colon, FITC)
gMFI CD3 live, singlet cells (colon)
*
*
CD3 (brain, FITC)
gMFI CD3 live, singlet cells (brain)
**
*
E
F
G
H
RORγt
RORγt (colon, PE)
gMFI RORγt live, singlet cells (colon)
0.085
0.085
RORγt (brain, PE)
gMFI RORγt live, singlet cells (brain)
ns
*
I
J
K
L
IL-17A
IL-17A (colon, PE-cy7)
IL-17A live, singlet cells (colon)
IL-17A (brain, PE-cy7)
gMFI IL-17A live, singlet cells (brain)
ns
ns
M
N
O
P
IFN-γ
IFN-γ (colon, BV421)
IFN-γ live, singlet cells (colon)
IFN-γ (brain, BV421)
IFN-γ live, singlet cells (brain)
Media
E. lenta Δcgr
E. lenta wt

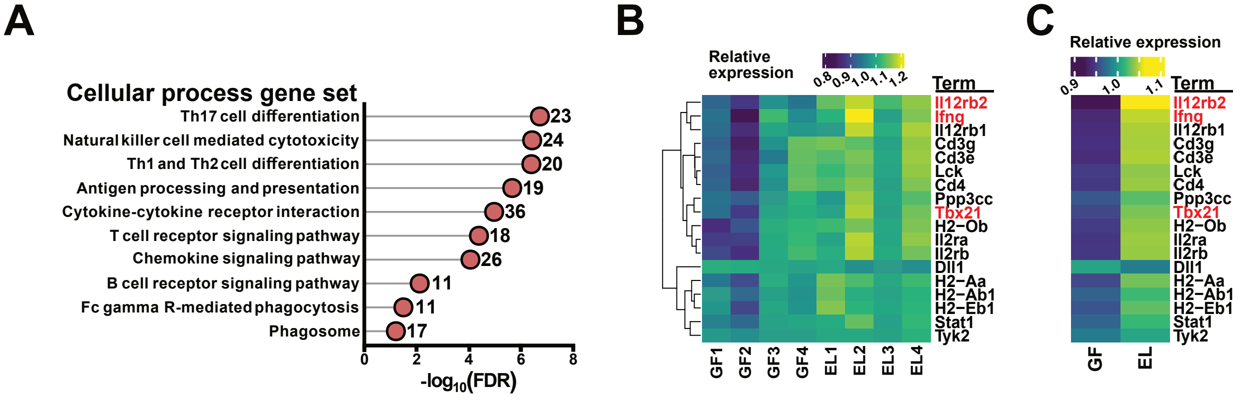
A
Cellular process gene set
Th17 cell differentiation
Natural killer cell mediated cytotoxicity
Th1 and Th2 cell differentiation
Antigen processing and presentation
Cytokine-cytokine receptor interaction
T cell receptor signaling pathway
Chemokine signaling pathway
B cell receptor signaling pathway
Fc gamma R-mediated phagocytosis
Phagosome
23
24
20
19
36
18
26
11
11
17
0 2 4 6 8
-log10(FDR)
B
Relative expression
0.8 0.9 1.0 1.1 1.2
Term
Il12rb2
Ifng
Il12rb1
Cd3g
Cd3e
Lck
Cd4
Ppp3cc
Tbx21
H2−Ob
Il2ra
Il2rb
Dll1
H2−Aa
H2−Ab1
H2−Eb1
Stat1
Tyk2
GF1 GF2 GF3 GF4 EL1 EL2 EL3 EL4
C
Relative expression
0.9 1.0 1.1
Term
Il12rb2
Ifng
Il12rb1
Cd3g
Cd3e
Lck
Cd4
Ppp3cc
Tbx21
H2−Ob
Il2ra
Il2rb
Dll1
H2−Aa
H2−Ab1
H2−Eb1
Stat1
Tyk2
GF EL

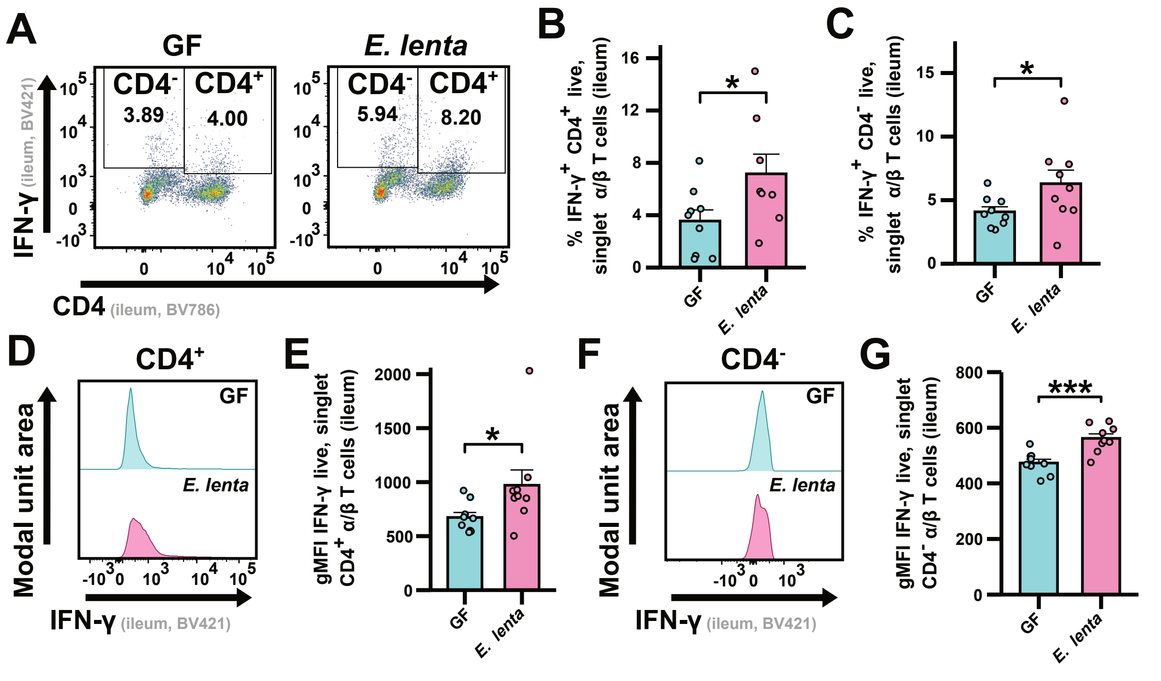

A
GF
E. lenta
CD4-
CD4+
3.89
4.00
5.94
8.20
IFN-γ (ileum, BV421)
CD4 (ileum, BV786)
B
% IFN-γ+ CD4+ live, singlet α/β T cells (ileum)
GF
E. lenta
*
C
% IFN-γ+ CD4- live, singlet α/β T cells (ileum)
*
D
CD4+
Modal unit area
IFN-γ (ileum, BV421)
E
gMFI IFN-γ live, singlet CD4+ α/β T cells (ileum)
*
F
CD4-
G
gMFI IFN-γ live, singlet CD4- α/β T cells (ileum)
***

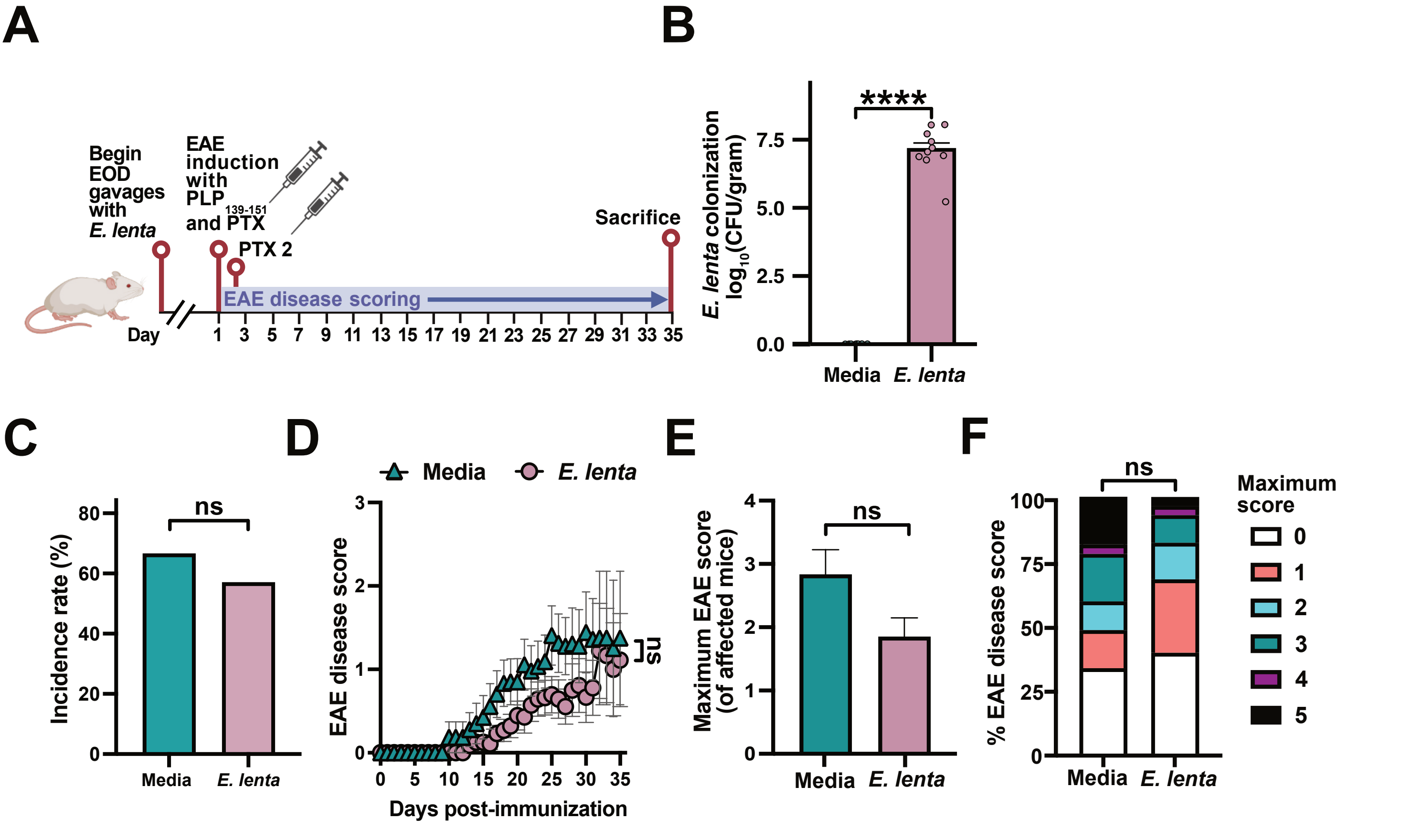
A
Begin EOD gavages with E. lenta
EAE induction with PLP 139-151 and PTX
PTX 2
Sacrifice
EAE disease scoring
Day
1 3 5 7 9 11 13 15 17 19 21 23 25 27 29 31 33 35
B
E. lenta colonization log10(CFU/gram)
****
Media
E. lenta
C
Incidence rate (%)
ns
Media
E. lenta
D
Media
E. lenta
EAE disease score
Days post-immunization
ns
E
Maximum EAE score (of affected mice)
ns
Media
E. lenta
F
% EAE disease score
ns
Media
E. lenta
Maximum score
0
1
2
3
4
5

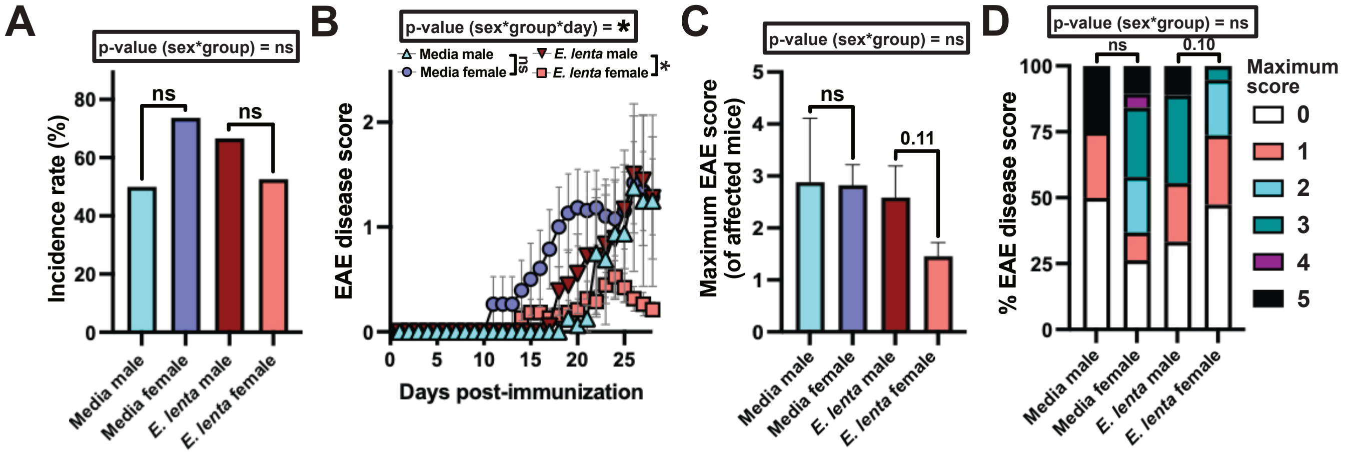

A
p-value (sex*group) = ns
Incidence rate (%)
ns
ns
Media male
Media female
E. lenta male
E. lenta female
B
p-value (sex*group*day) = *
Media male
Media female
ns
E. lenta male
E. lenta female
*
EAE disease score
Days post-immunization
C
p-value (sex*group) = ns
Maximum EAE score (of affected mice)
ns
0.11
D
p-value (sex*group) = ns
ns
0.10
% EAE disease score
Maximum score
0
1
2
3
4
5

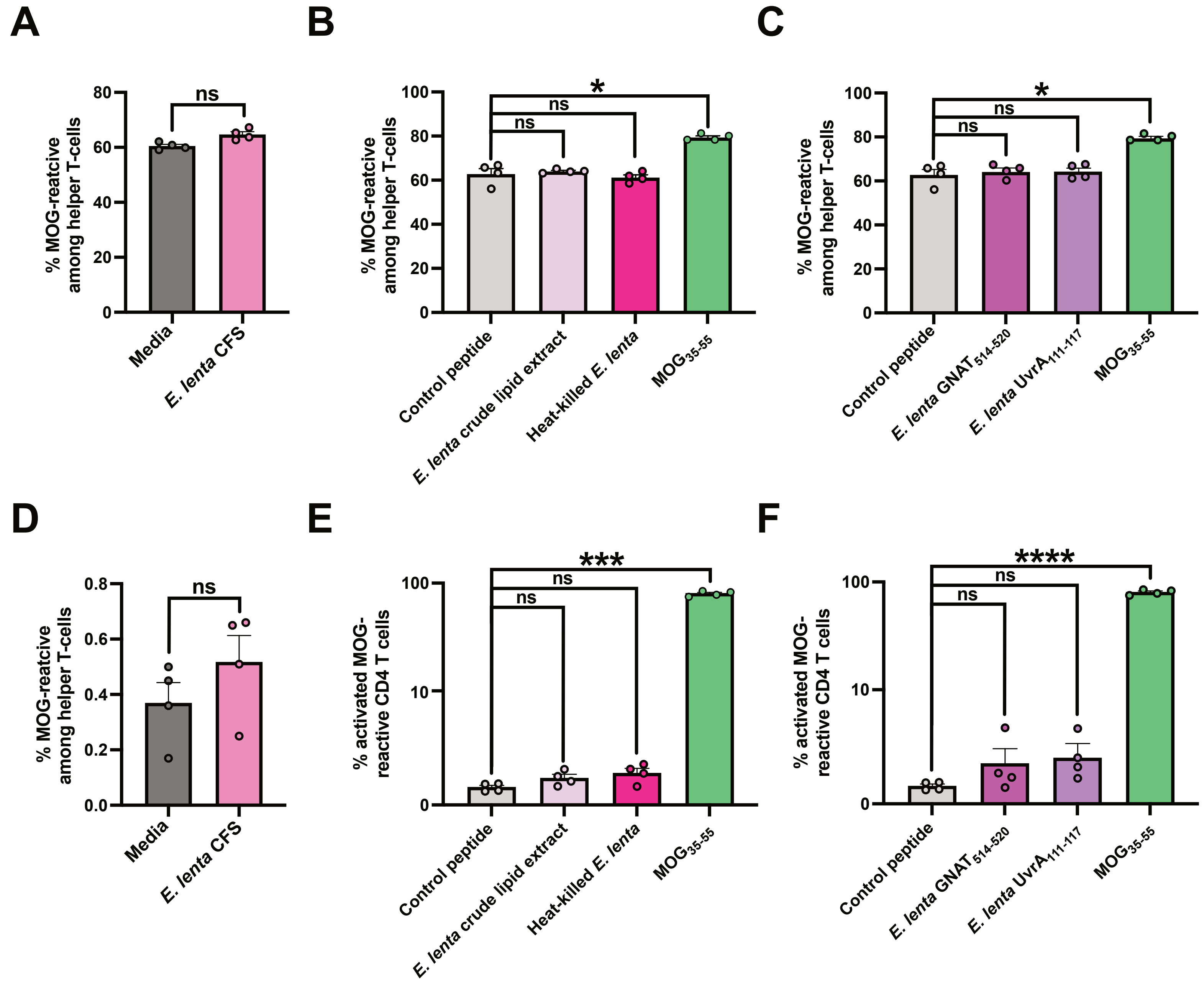
A
B
C
D
E
F
% MOG-reatcive among helper T-cells
% activated MOG-reactive CD4 T cells
ns
*
***
****
Media
E. lenta CFS
Control peptide
E. lenta crude lipid extract
Heat-killed E. lenta
MOG35-55
E. lenta GNAT514-520
E. lenta UvrA111-117

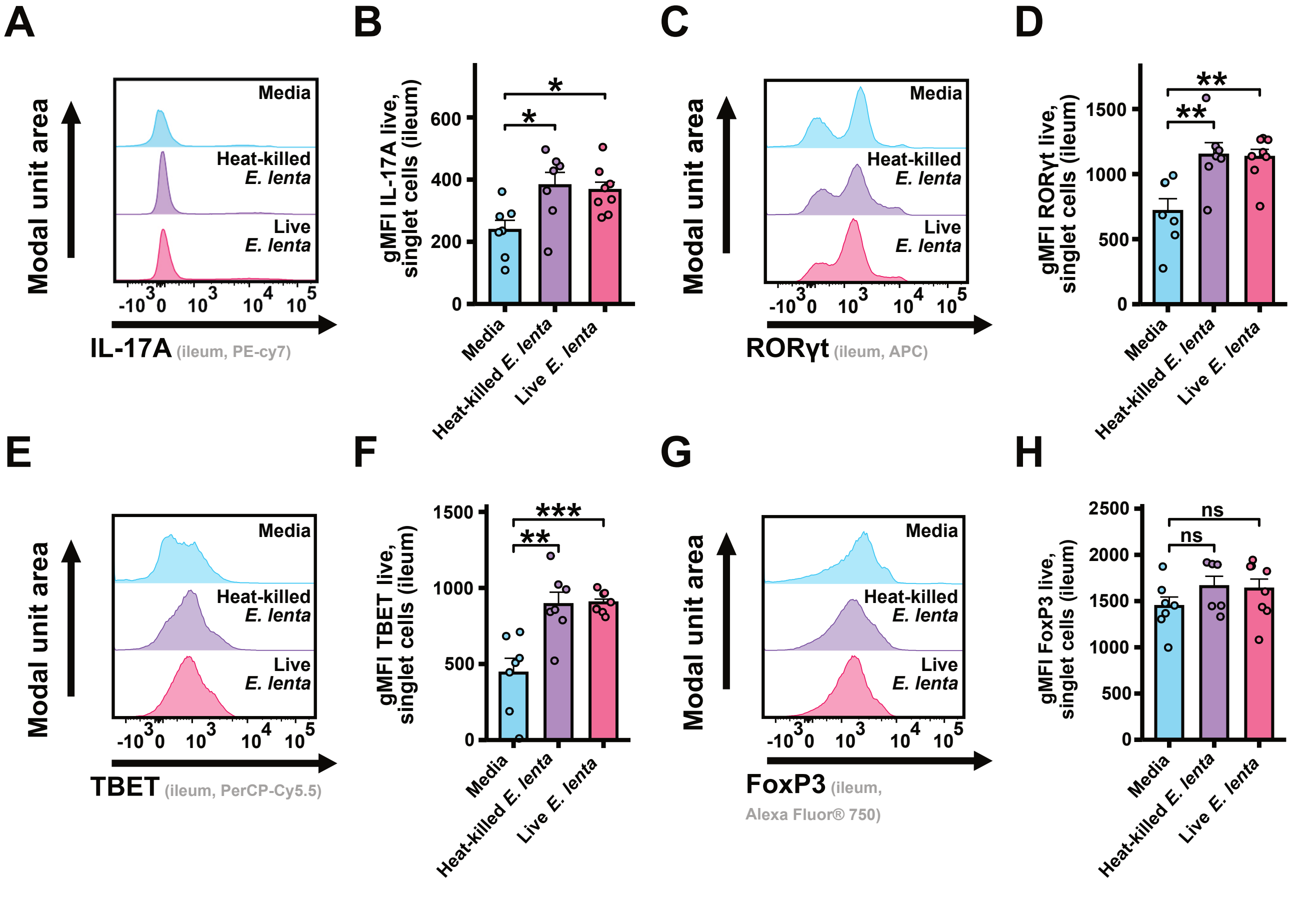
A
Modal unit area
Media
Heat-killed E. lenta
Live E. lenta
-10^3 0 10^3 10^4 10^5
IL-17A (ileum, PE-cy7)
B
gMFI IL-17A live, singlet cells (ileum)
0
200
400
600
*
*
Media
Heat-killed E. lenta
Live E. lenta
C
Modal unit area
Media
Heat-killed E. lenta
Live E. lenta
-10^3 0 10^3 10^4 10^5
RORγt (ileum, APC)
D
gMFI RORγt live, singlet cells (ileum)
0
500
1000
1500
**
**
Media
Heat-killed E. lenta
Live E. lenta
E
Modal unit area
Media
Heat-killed E. lenta
Live E. lenta
-10^3 0 10^3 10^4 10^5
TBET (ileum, PerCP-Cy5.5)
F
gMFI TBET live, singlet cells (ileum)
0
500
1000
1500
***
**
Media
Heat-killed E. lenta
Live E. lenta
G
Modal unit area
Media
Heat-killed E. lenta
Live E. lenta
-10^3 0 10^3 10^4 10^5
FoxP3 (ileum, Alexa Fluor® 750)
H
gMFI FoxP3 live, singlet cells (ileum)
0
500
1000
1500
2000
2500
ns
ns
Media
Heat-killed E. lenta
Live E. lenta

bioRxiv preprint doi: https://doi.org/10.64898/2026.03.16.711194; this version posted March 23, 2026. The copyright holder for this preprint (which was not certified by peer review) is the author/funder, who has granted bioRxiv a license to display the preprint in perpetuity. It is made available under aCC-BY 4.0 International license.

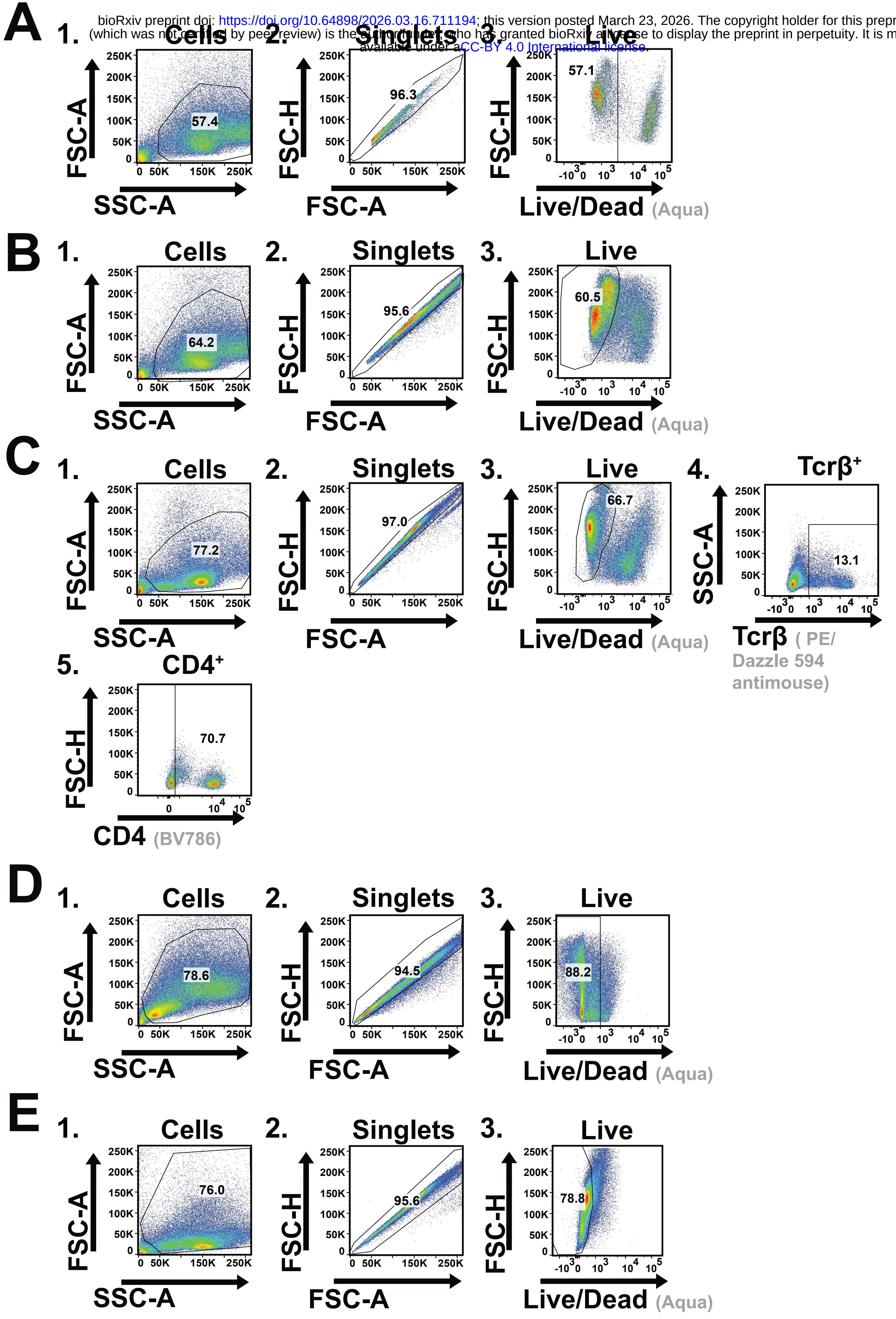

Supplement: Supplement 4 — Figure S1. Meta-analysis reveals sex-associated gut bacterial species, related to Figure 1. (A) Differentially prevalent microbial species by sex in the curatedMetagenomicData database (dashed line indicates FDR < 0.05) determined via FDR-adjusted logistic models (glm(species prevalence ~ sex + age + BMI + continent + health + median read length + sequencing depth + DNA extraction kit, family=binomial)). (B, C) Top hits using a stricter cutoff (dashed lines indicate lowest quintile FDR and highest quintile effect size) for (B) prevalence and (C) abundance. (B) Logistic model with with sex, age, BMI, continent, health, and sequencing metrics as fixed effects (species prevalence ~ sex + age + BMI + continent + health + median read length + sequencing depth + DNA extraction kit, family=binomial). (C) MaAsLin2 model with sex, age, BMI, continent, health, and sequencing metrics as fixed effects and study as a random effect (CLR-normalized relative abundance ~ sex + age category + BMI category + continent + health category + median read length category + sequencing depth category + DNA extraction kit category + (1∣study)). (D) Venn diagrams display the top sex-associated bacterial species common to both (B) prevalence and (C) abundance models. (A-C) Each dot represents one species, colored by sex of enrichment.(A) Differentially prevalent microbial species by sex in the curatedMetagenomicData database (dashed line indicates FDR < 0.05). (B, C) Top hits using a stricter cutoff (dashed lines indicate lowest quintile FDR and highest quintile effect size) for (B) prevalence and (C) abundance. (D) Venn diagrams displayed the top sex-associated bacterial species common to both (B) prevalence and (C) abundance models. (A-C) Each dot represents one species, colored by sex of enrichment. Statistics: (A, B) FDR-adjusted logistic model (glm(species prevalence ~ sex + age + BMI + continent + health + median read length + sequencing depth + DNA extraction kit, family=binomial), STAR M [file NIHPP2026.03.16.711194v2-supplement-4.pdf]
